# Experimental and histological evaluation of different clamp technique for pulmonary artery

**DOI:** 10.1093/icvts/ivad119

**Published:** 2023-07-31

**Authors:** Yoshiki Chiba, Yuki Takahashi, Yoshiaki Takase, Kodai Tsuruta, Ryunosuke Maki, Masahiro Miyajima, Hirofumi Ohnishi, Atsushi Watanabe

**Affiliations:** Department of Thoracic Surgery, Sapporo Medical University School of Medicine and Hospital, Sapporo, Japan; Department of Thoracic Surgery, Sapporo Medical University School of Medicine and Hospital, Sapporo, Japan; Department of Thoracic Surgery, Sapporo Medical University School of Medicine and Hospital, Sapporo, Japan; Department of Thoracic Surgery, Sapporo Medical University School of Medicine and Hospital, Sapporo, Japan; Department of Thoracic Surgery, Sapporo Medical University School of Medicine and Hospital, Sapporo, Japan; Department of Thoracic Surgery, Sapporo Medical University School of Medicine and Hospital, Sapporo, Japan; Department of Basic Medical Science and Department of Public Health, Sapporo Medical University, Sapporo, Japan; Department of Thoracic Surgery, Sapporo Medical University School of Medicine and Hospital, Sapporo, Japan

**Keywords:** Burst pressure, Clamp pressure, Double-loop technique, Histological damage, Main pulmonary artery, Minimally invasive surgery

## Abstract

**OBJECTIVES:**

The double-loop technique has been used in our clinical settings for pulmonary arterioplasty and/or injured artery repair during thoracoscopic anatomical lung resection. We evaluated the pressure resistance capacity and intimal load to determine the effectiveness and safety of the double-loop technique.

**METHODS:**

The double-loop technique, DeBakey clamp, Fogarty clamp, endovascular clips and vessel loop technique were evaluated. During an experimental study, a polyvinyl alcohol main pulmonary artery model, manometer and in-deflation device were used to measure the burst pressure. The maximum clamp pressure was measured using a pressure-measuring film. Each measurement was performed 10 times. During the histological study, we measured the burst pressure and evaluated the intimal damage of the human pulmonary artery associated with the double-loop technique and DeBakey clamp.

**RESULTS:**

The experimental burst pressure (mmHg) and maximum clamp pressure (MPa) between the double-loop technique and DeBakey at the third notch were not significantly different (24.6 ± 2.8 and 21.8 ± 2.8, *P *=* *0.094; 1.54 ± 0.12 and 1.49 ± 0.12, *P *=* *0.954). During the histological study, the burst pressures of the double-loop technique and DeBakey at the third notch were also not significantly different (*P *=* *0.754). Furthermore, the double-loop technique resulted in only intimal deformation in each five samples.

**CONCLUSIONS:**

The double-loop technique is feasible for thoracoscopic anatomical lung resection because it has similar pressure resistance capacity and intimal load as DeBakey at the 3rd notch.

**Figure:**
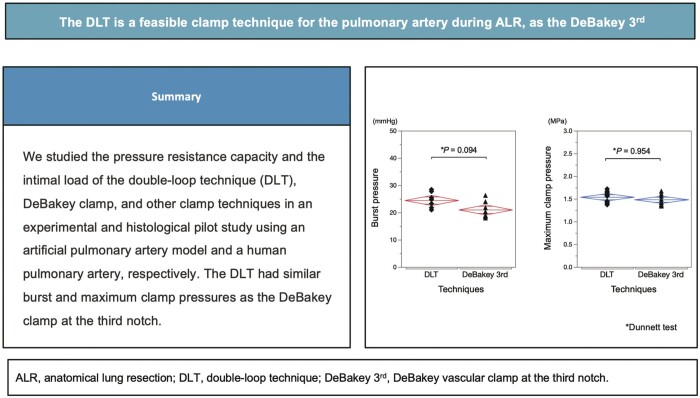

## INTRODUCTION

Pulmonary artery clamping using a vascular clamp on an extended incision or an open conversion has traditionally been the gold standard for patients with intraoperative bleeding or severe adhesions during thoracoscopic anatomical lung resection (ALR) [1, 2]. However, the clamp technique should be practicable and safe for use in the thoracic cavity, and it needs to accommodate the recent developments in minimally invasive surgery. We previously reported the double-loop technique (DLT) as a possible clamping method in thoracoscopic ALR [3] and demonstrated that the DLT had the following advantages: it does not disturb the thoracoscopic view; an extended incision or an additional incision is not necessary for clamping; specific devices or techniques are not required; and it is inexpensive. Furthermore, we have performed the DLT for 51 patients during ALR at our institute and observed its benefits of minimal slipping and acceptable partial remnants of silk sutures. Several studies have suggested using clamp techniques during thoracoscopic ALR such as the DeBakey clamp, Fogarty clamp, endoscopic vascular clamp and vessel loop technique (VLT) [4–6]. However, the safety and effectiveness of these widely used techniques have not been objectively clarified, and the pressure resistance capacity and intimal load of the pulmonary artery have not been evaluated. Although several previous animal studies have used vascular clamps on the aortas, carotid arteries and femoral arteries, the results are not considered applicable to the pulmonary artery, which is part of the low-pressure circulatory system [7–9]. However, the histological analysis of the effectiveness and safety of each clamp technique for the human main pulmonary artery is quite difficult. We believe that using cadavers or human lung specimens undergoing pneumonectomy is the only way; however, these studies have significant limitations. A cadaveric clamp study is performed after formalin fixation; therefore, the actual histological changes cannot be evaluated even if the clamp techniques are performed. In a study using human lung specimens undergoing pneumonectomy, it was difficult to accumulate samples because early diagnosis and treatment of lung cancer have become possible recently. Therefore, we initially evaluated the effectiveness of the DLT and other clamp techniques by measuring the pressure resistance capacity and the intimal load of the pulmonary artery using an artificial vessel model and pressure-measuring film. Furthermore, the pilot data of our ongoing histological study using human pulmonary arteries are also presented to demonstrate the safety of DLT.

## MATERIALS AND METHODS

### Ethical statement

The Sapporo Medical University Hospital Institutional Review Board approved this experimental and histological studies on 12 August 2021 (approval number: 312-222). Individual informed consent was waived in the experimental study, whereas, the histological study required it and we obtained written consent forms from all patients.

### Experimental study

#### Study design

**Figure 1:**
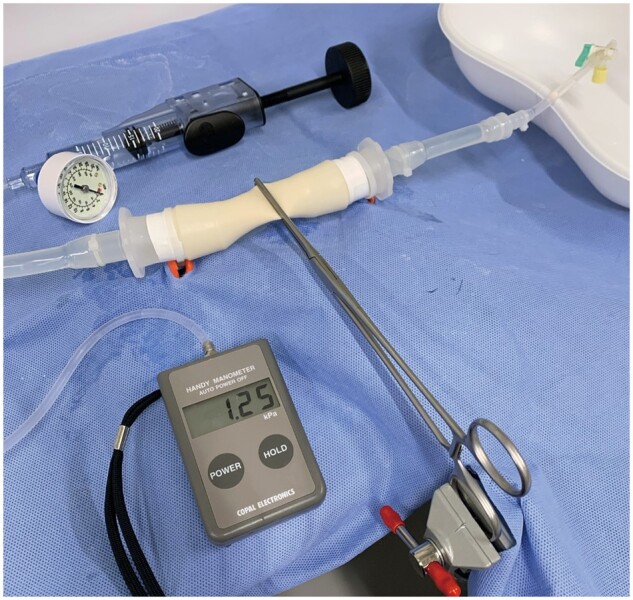
During the experimental study, intravascular pressure was measured using a connected manometer and an in-deflation device.

We used an artificial main pulmonary artery model (diameter, 20 mm; thickness, 1 mm) that was constructed of polyvinyl alcohol (Wetlab Co., Osaka, Japan). The vessel model has been used as a cerebral artery model for monitoring blood flow and preoperative simulations [10, 11]. The main pulmonary artery model was fixed to a stand and filled with normal saline using the connected tube and stopcock. Next, the intravascular pressure was measured using a connected manometer (PG-100; Nidec Copal Electronics, Tokyo, Japan) and maintained at 15 mmHg while each clamp technique was applied (Fig. 1). Then, we gradually pressurized the main pulmonary artery model with a connected in-deflation device to measure the burst pressure. All experiments were performed in a room with a temperature of 24°C and humidity of 58%.

#### Clamp techniques

**Figure 2:**
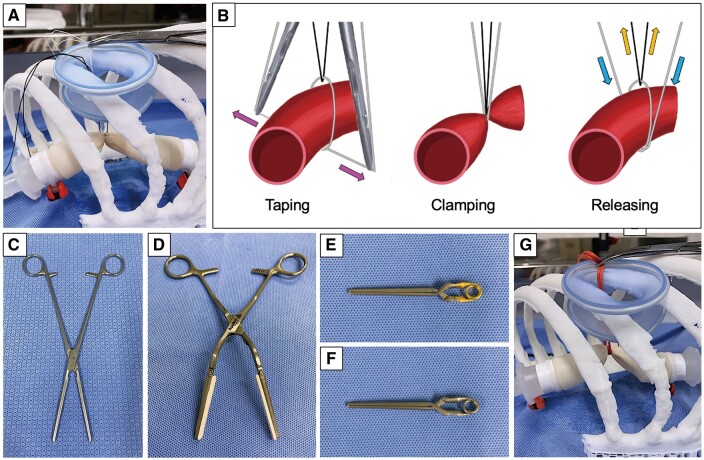
Clamp techniques. (**A**) Double-loop technique. (**B**) Double-loop technique procedures. A 1–0 silk suture (grey) encircled the pulmonary artery twice to clamp it, and another 1–0 silk suture (black) was threaded through the circle to release the suture clamp. The grey 1–0 silk suture was drawn horizontally (pink arrow) and affixed to the chest wall. The black silk suture through the circle was pulled outside (orange arrow) to easily release the suture clamp (blue arrow). (**C**) DeBakey vascular clamp. (**D**) Fogarty vascular clamp. (**E**) Endoscopic vascular clip with a closing pressure of 300 g (gold clip). (**F**) Endoscopic vascular clip with a closing pressure of 450 g (silver clip). (**G**) Vessel loop technique.

DeBakey vascular clamp (FB-454R; Aesculap AG & Co., Tokyo, Japan) and Fogarty vascular clamp with an angled handle (model A 3204; Applied Medical Resources, Santa Margarita, CA, USA) were used for this study (Fig. 2C and D). The DeBakey and Fogarty clamps were fixed to a vice after clamping to minimize the workforce. The main pulmonary artery model was clamped using the DeBakey clamp at the third notch (DeBakey 3rd), DeBakey clamp at the fourth notch (DeBakey 4th), Fogarty clamp at the first notch (Fogarty 1st), and Fogarty clamp at the second notch (Fogarty 2nd). We also used two endoscopic vascular clips with closing pressures of 300 g (gold clip) and 450 g (silver clip), respectively (PL543S and PL545S; Aesculap AG & Co.) (Fig. 2E and F).

We created a left thorax (actual-size) model of acrylonitrile butadiene styrene filament using a three-dimensional printer (UP BOX+; PP3DP, Tokyo, Japan) using clinical data of digital imaging of a patient with an average Japanese physique. The thorax model was placed with its outer surface at 10 cm from the main pulmonary artery model, thus replicating the distance in the actual thorax. A lap protector mini (Hakko Co., Ltd. Medical Device Division, Tokyo, Japan) was placed on the fourth intercostal anterior axillary line; this procedure was similar to that performed in clinical practice at our institute. The thorax model was used to fix silk or vessel loops outside the thorax using a lap protector mini and mosquito clamp. Similar to our previous study, the DLT was performed using two 1–0 silk sutures [3]. The main pulmonary artery model was encircled twice with a 1–0 silk suture (white), and another 1–0 silk suture (black) was threaded through the circle to release the suture clamp. The encircling 1–0 suture was drawn horizontally through the thorax model with thoracoscopic forceps and fixed outside the thorax with a mosquito clamp while the tension was maintained (Fig. 2A and B). We used a silicone rubber vessel loop (42 cm, model 1001–76; Surg-I-Loop, Scanlan International, St. Paul, MN, USA) to enforce the double-loop vascular sling technique. The vessel loop was drawn horizontally and fixed outside the thorax model, similar to the DLT (Fig. 2G) [4]. For the DLT and VLT, a pilot experiment was conducted to measure the burst pressure five times for each clamp technique. The silk sutures and vessel loops were marked at a median fixed position (specific position) where they tolerated the main pulmonary artery pressure of 20 mmHg, which was the outcome of the pilot experiment.

#### Outcomes measured

We used three indicators to validate the pressure-resistance capacity and intimal load on the pulmonary artery for each clamp technique: burst pressure, traction force and maximum clamp pressure. Each measurement was performed 10 times for each clamp technique and the mean values were compared between the groups. Additionally, we homogenized the DLT and VLT by performing traction and fixation at each specific position obtained during the pilot experiment while measuring the burst pressure and maximum clamp pressures.

##### Burst pressure

Burst pressure was defined as the manometer pressure at the time of leakage of normal saline from the end of the connected tube after clamping the main pulmonary artery model and gradually applying pressure using an in-deflation device.

##### Traction force

The traction forces of the DLT and VLT were measured using an electronic scale (WH-A08; Weiheng, Guangdong, China), and the mean values were calculated (Supplementary Material, Fig. S1). Additionally, the VLT was used to measure the pressure at the same specific position as the DLT to evaluate the pressure at the same traction force as the DLT.

##### Maximum clamp pressure

**Figure 3:**
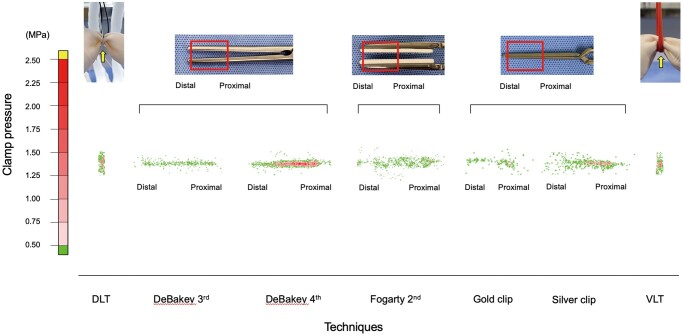
Measuring clamp pressure (MPa) using a pressure image analysis system. During the DLT and VLT, 2-mm-wide film was cut and placed under the pulmonary artery model (yellow arrow). Red rectangles depict the clamping areas of the forceps and clips, whereas ‘distal’ and ‘proximal’ indicate the distance from the hinges of the forceps and clips. DeBakey 3rd: DeBakey vascular clamp at the third notch; DeBakey 4th: DeBakey vascular clamp at the fourth notch; DLT: double-loop technique; Fogarty 2nd: fibrous jaw clamp at the second notch; VLT: vessel loop technique.

The intimal load of the pulmonary artery caused by clamping was evaluated by measuring the maximum clamp pressure using pressure-measuring film (Prescale LLLW; Fujifilm, Tokyo, Japan) that had been used for an aortic occlusion experimental study of pigs [7] and for measuring the tip pressure of laparoscopic grasping forceps during previous studies [12]. Pressure measurements of the DeBakey clamp, Fogarty clamp and vascular clips were obtained by placing the film between the clamping position, as previously performed by Rylski *et al.* [7]. Measurements during the DLT and VLT were obtained by inserting a 2-mm-wide film under the main pulmonary artery model, which was inferred to have a stronger load. Each fixation was performed at a specific position that could tolerate 20 mmHg of pressure resistance. Then, the films were scanned and the clamp pressure values were determined using a pressure image analysis system (FPD-8010 J; Fujifilm) (Fig. 3). Continuous pressure was applied with a 15-min clamp duration. Experiments were repeated 10 times for each clamp technique. The maximum clamp pressure was defined as the maximum value according to the pressure image analysis system, and the mean values were compared. Additionally, clamp pressures of the DeBakey clamp, the Fogarty clamp, and vascular clips were measured at the proximal and distal points relative to the hinges of the forceps or clips.

### Histological study

#### Study design

**Figure 4:**
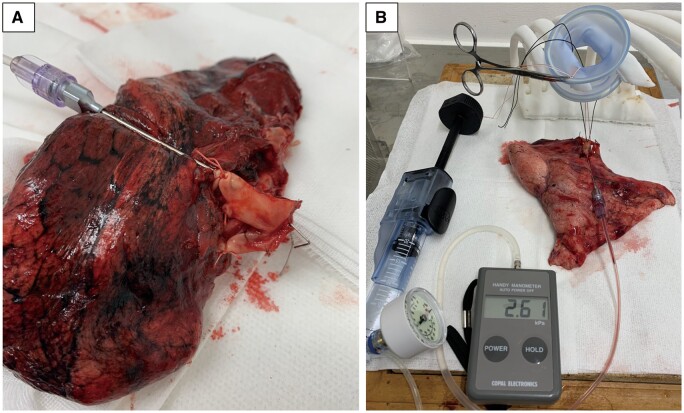
The histological study. (**A**) A heparin needle was inserted in the peripheral pulmonary artery and all other peripheral branches were ligated. (**B**) The double-loop technique. Pressurizing and measuring were performed in the same methods as that during the experimental study.

We used human pulmonary arteries from resected specimens of the lobe of the lung after lobectomy for lung cancer. A heparin needle was inserted in the peripheral pulmonary artery, and all other peripheral branches were ligated (Fig. 4A). The clamp techniques used for each pulmonary artery were randomly allocated. After each clamp technique was performed, the staple used for pulmonary artery transection was removed and evaluated for leakage. Pressurization and measurements were performed in the same methods as during experimental study. The pulmonary artery diameter was measured as the outer diameter of the vessel at a pressure of 15 mmHg. During this histological study, pulmonary arteries with an external diameter >10 mm were included. A pilot experiment involving the DLT was also conducted to measure the burst pressure five times as in the experimental study and the silk sutures were marked at a specific position. After a clamp duration of 15 min, clamp sections were cut into 7-mm pieces and fixed with 10% formalin neutral buffer solution. Samples obtained for histological evaluation were stained with Elastica Van Gieson stain. The expected sample size was 10 samples for each technique; however, this was not achieved at present. To evaluate the safety, five samples for each technique (DLT, DeBakey 3rd, and DeBakey 4th) were assessed, and the results are presented as pilot data.

#### Outcomes measured

##### Burst pressure

Burst pressure was measured in the same methods as it was during the experimental study and by modifying the experimental model of Tsunezuka *et al.* (Fig. 4B) [13].

##### Histological findings

Furthermore, we evaluated the 2 mm or more intimal denudation of the clamped sections of pulmonary arteries with reference to Zhang’s score [14]. Normal pulmonary artery sections were evaluated as controls. If the controls experienced damage due to surgical procedures, then all samples from the same patient were excluded.

### Statistical analysis

Continuous variables are expressed as the mean and standard deviation. Categorical variables are expressed as numbers. The normality of continuous variables was evaluated using the Shapiro–Wilk test. We confirmed that all continuous variables satisfied the normal assumption. The appropriate sample size was calculated for a statistical power of 0.8, a significance level of 0.05, and an effect size of 0.3; therefore, 10 samples in each group were required because of slight variability. A one-way analysis of variance was performed, followed by the Dunnett test as a post-hoc test for the analysis of continuous variables to compare the burst pressure, traction force and maximum clamp pressure between the DLT as the reference and each clamp technique. Additionally, regarding the vascular clamps and clips, a comparison of maximum clamp pressure variables of the distal and proximal regions was performed using the paired *t*-test. The Wilcoxon signed-rank test was performed to compare the burst pressures of human pulmonary arteries between the DLT and DeBakey clamp. *P* values of 0.05 or less were considered significant. Statistical analyses were performed using JMP Pro 15 (SAS Inc., Cary, NC, USA).

## RESULTS

**Figure 5:**
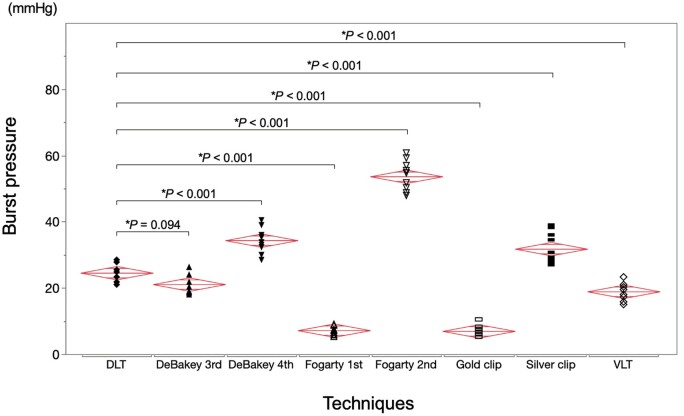
Comparison of the burst pressures associated with the double-loop technique and other clamp techniques. DeBakey 3rd: DeBakey vascular clamp at the third notch; DeBakey 4th: DeBakey vascular clamp at the fourth notch; DLT: double-loop technique; Fogarty 1st: fibrous jaw clamp at the first notch; Fogarty 2nd: fibrous jaw clamp at the second notch; VLT: vessel loop technique. *Dunnett test

Burst pressure associated with the DLT as the reference was compared with that of each clamp technique (Fig. 5). The mean burst pressures (mmHg) were 24.6 ± 2.8 with the DLT, 21.8 ± 2.8 with DeBakey 3rd, 34.4 ± 3.7 with DeBakey 4th, 7.2 ± 1.4 with Fogarty 1st, 53.7 ± 4.5 with Fogarty 2nd, 7.0 ± 1.5 with the gold clip, 31.8 ± 4.7 with the silver clip, 18.9 ± 2.6 with the VLT. No significant difference was found between the DLT and DeBakey 3rd; however, DeBakey 4th had a significantly higher pressure than the DLT (*P *=* *0.094 and *P *<* *0.001). Furthermore, the DLT was associated with significantly higher pressures than Fogarty 1st, the gold clip, and the VLT (*P *<* *0.001 for these three techniques). In contrast, Fogarty 2nd and the silver clip had significantly higher pressures than the DLT (*P *<* *0.001 for both).

Traction forces associated with the DLT and VLT are presented in Supplementary Material, Fig. S2A. The mean traction forces (kg) were 0.12 ± 0.02 with the DLT and 0.30 ± 0.03 with the VLT. The DLT had a lower traction force than the VLT (*P *<* *0.001). The burst pressure outcome of the VLT fixed at the specific position of the DLT is presented in Supplementary Material, Fig. S2B. The mean burst pressure (mmHg) was 9.4 ± 1.4 with the VLT; this value was significantly lower than that associated with the DLT (*P *<* *0.001).

**Figure 6:**
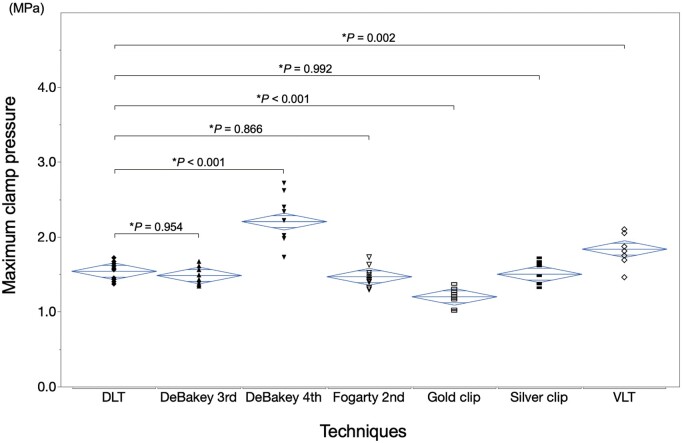
Comparison of maximum clamp pressures at 15 min for all clamp techniques. DeBakey 3rd: DeBakey vascular clamp at the third notch; DeBakey 4th: DeBakey vascular clamp at the fourth notch; DLT: double-loop technique; Fogarty 2nd: fibrous jaw clamp at the second notch; VLT: vessel loop technique. *Dunnett test.

The maximum clamp pressure among all techniques using a 15-min clamp duration is presented in Fig. 6. The mean maximum clamp pressures (MPa) were 1.54 ± 0.12 with the DLT, 1.49 ± 0.12 with DeBakey 3rd, 2.21 ± 0.31 with DeBakey 4th, 1.47 ± 0.13 with Fogarty 2nd, 1.20 ± 0.11 with the gold clip, 1.50 ± 0.15 with the silver clip and 1.84 ± 0.21 with the VLT. No significant difference in the maximum clamp pressure between the DLT and DeBakey 3rd, Fogarty 2nd or the silver clip (*P *=* *0.954, 0.866 and 0.992) was observed. The gold clip was associated with significantly lower pressure than the DLT (*P *<* *0.001). In contrast, DeBakey 4th and the VLT had significantly higher values than the DLT (*P *<* *0.001 and 0.002).

Comparisons of the maximum clamp pressures at the distal and proximal points with the forceps or clips are presented in Supplementary Material, Fig. S3. For all vascular clamps without the Fogarty clamp, the pressures in the proximal region were significantly higher than those in the distal region.

Characteristics of patients included in the histological study are summarized in Table 1. The results of 10 patients and 15 sections of the pulmonary artery were included as pilot data. A comparison of burst pressure of the DLT and DeBakey 3rd during the histological study is presented in Supplementary Material, Fig. S4. The mean traction force (kg) was 0.044 ± 0.004 with the DLT. The median burst pressures (mmHg) were 69.0 (range, 54.8–92.3) with the DLT, 62.3 (range, 57.0–94.5) with DeBakey 3rd. A significant difference between the DLT and DeBakey 3rd was not observed (*P *=* *0.754). DeBakey 4th was associated with pressure resistance capacity of more than 150 mmHg in all samples.

**Table 1:** Patient characteristics: histological study

	*n* = 10
Male/female	7/3
Age (years), median (range)	71 (58–83)
BMI, median (range)	24.0 (19.1–33.9)
Pulmonary hypertension	0
Lobe	10
Right	4
Left	6
Tumour size (mm), median (range)	29 (11–56)
Histology	
Adenocarcinoma	5
Squamous cell carcinoma	4
Small cell carcinoma	1
Pathological stage^*^	
IA/IB	4/3
IIA/IIB	0/1
IIIA/IIIB	1/0
IV	1
Clamped pulmonary artery sections	15
Right	
A^8 + 9+10^	3
A^9 + 10^	1
Left	
A^8 + 9+10^	5
A^9 + 10^	4
A^8^	2

* Pathological stage classified according to the 8th UICC TNM classification.

**Figure 7:**
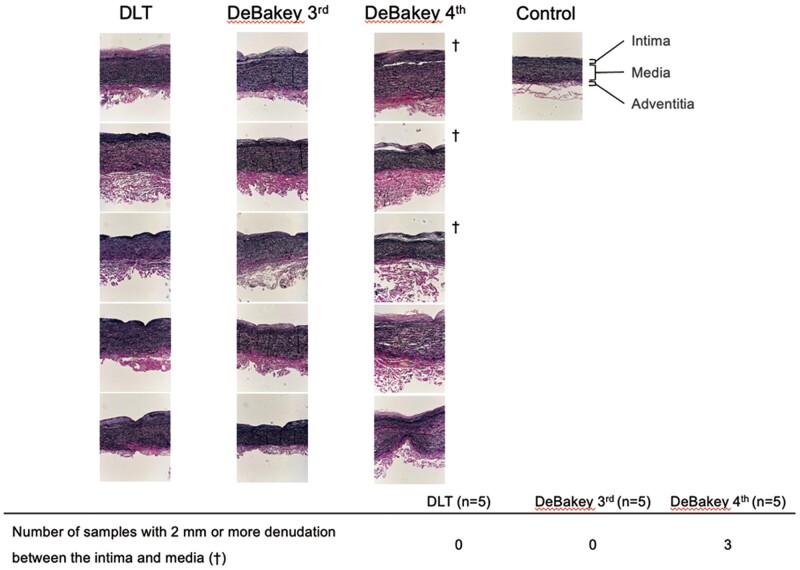
Histological findings of pulmonary arteries with clamping using the DLT, DeBakey 3rd, and DeBakey 4th. The clamped sections of the pulmonary artery were stained with EVG (×100). ^†^Number of samples with 2 mm or more denudation between the intima and media. DeBakey 3rd: DeBakey vascular clamp at the third notch; DeBakey 4th: DeBakey vascular clamp at the fourth notch; DLT: double-loop technique.

The histological findings of 15 min of clamping during the DLT, DeBakey 3rd, and DeBakey 4th are presented in Fig. 7. The DLT and DeBakey 3rd had no 2 mm or more intimal denudation; however, DeBakey 4th had three samples with it.

## DISCUSSION

This study is the first to evaluate the pressure resistance capacity and clamp pressure associated with the available clamp techniques for thoracoscopic ALR using the main pulmonary artery model, pressure-measuring film and pressure image analysis system. Knowledge of the characteristics of each clamp technique for the pulmonary artery is important before conducting the histological study because of its thin and soft vessel wall. Furthermore, this is the first histological study to use human pulmonary arteries to evaluate the intimal damage with each clamp technique; however, it is still ongoing. Therefore, we believe that both studies are valuable.

Our studies demonstrated that the DLT is similar to DeBakey 3rd in the burst and maximum clamp pressures and intimal load of the human pulmonary artery. Whereas the DLT was inferior to DeBakey 4th in the burst pressure; however, DeBakey 4th had significantly higher maximum clamp pressure than the DLT, and the histological study demonstrated that intimal denudation occurred in three of the five samples. Therefore, DeBakey 4th might be associated with over-pressurization and a high intimal load in the context of pulmonary artery blood pressure, which is normally lower than systemic arterial blood pressure. To adjust the clamp pressure and decrease pulmonary artery overload during the DLT, the 1–0 silk suture was drawn horizontally through the thorax and affixed outside the thorax while maintaining tension but not too much traction. The DLT has sometimes been avoided for clinical cases because of concerns regarding its untested safety. However, our study shows that the DLT is a feasible technique because it is demonstrated that the potential intimal load is equal to that of DeBakey 3rd.

Vascular clamp forceps are generally used during thoracic surgery; however, extending the incision or placing an additional incision is necessary to avoid interfering with the thoracoscopic view [2, 15, 16]. Furthermore, vascular clamp jaws with unequal pressure distribution between the proximal and distal regions of the clamp might cause vessel injury [7]. Using the Fogarty clamp, which has a soft inserted surface, might reduce the variations in the pressure proximal and distal to the hinge, allowing for a clamp with a less intimal load on the vessels [7]; we noted similar results in our study. In contrast, the DLT does not obstruct the thoracoscopic view, requires no extension or addition of an incision, no specific device, and has no difference in pressure distribution. Therefore, we believe that the DLT is advantageous.

The endoscopic vascular clip has been feasible for clamping the main pulmonary artery during ALR [5]. In this study, the gold clip was inferior to the DLT, whereas the silver clip had significantly higher burst pressure and similar maximum clamp pressure compared to the DLT. These results might suggest that the silver clip was more suitable for clamping the main pulmonary artery. Additionally, the vascular clip had the greatest jaw force at the proximal position [6]; we noted comparable results. However, the use of clips requires special equipment, such as internal organ retractors, and fine adjustment of the clamp pressure cannot be performed with clips. Furthermore, endoscopic vascular clips have the risk of slippage and re-clamping is difficult in the thoracoscopic view.

The DLT and VLT are clamp techniques for thoracoscopic ALR that can be performed without extension of the incision or an additional incision [3, 4]. The vessel loop has generally been used as a sling or for clamping because it places a lower intimal load on the pulmonary artery [4]. However, the VLT had a significantly lower burst pressure and higher maximum clamp pressure than the DLT. Furthermore, the VLT requires stronger traction to achieve an effective clamp, most likely because of the silicon rubber vessel loop. The DLT using silk sutures does not require a strong traction force because the horizontal frictional force of the silk makes it difficult to achieve slack. Compared with the VLT, the DLT had important advantages such as minimal slippage, acceptable partial remnants and an unobstructed thoracoscopic view.

### Limitations

Consequential variations were possible when performing the DLT and VLT. To limit these as much as possible, we performed a single experiment using these techniques. Additionally, these techniques were made more uniform by marking the encircling silk suture and measuring the traction using an electronic scale. There might have been discrepancies in the burst and clamp pressures of the model and those of a real pulmonary artery. Normal saline is less viscous than human blood; however, the difference in the viscosity of normal saline and human blood is slight; therefore, the impact of this difference on these results is considered minimal. Despite using the largest-diameter pulmonary arteries available from the lobectomy specimen, these diameters were smaller than those of the main pulmonary artery. Therefore, the DLT traction force during the histological study was smaller than that during the experimental study, and the burst pressure during the histological study was larger than that during the experimental study.

## CONCLUSIONS

The DLT is a feasible technique for thoracoscopic ALR. The DLT had pressure resistance capacity and intimal load similar to those of DeBakey 3rd; therefore, the advantages of the DLT for thoracoscopic ALR should be recognized. Additional histological studies comprising larger sample sizes, the endoscopic vascular clips and VLT are necessary and underway at our institute.

## Supplementary Material

ivad119_Supplementary_Data

## Data Availability

Data will be shared on request to authors.

## Notes

Presented at the 75th Annual Scientific Meeting of the Japanese Association for Thoracic Surgery in Yokohama, Japan, October 5, 2022.

## ABBREVIATIONS

ALR: Anatomical lung resection
DeBakey 3rd: DeBakey vascular clamp at the third notch
DeBakey 4th: DeBakey vascular clamp at the fourth notch
DLT: Double-loop technique
Fogarty 1st: Fogarty vascular clamp at the first notch
Fogarty 2nd: Fogarty vascular clamp at the second notch
VLT: Vessel loop technique
